# Genome-wide identification and characterization of GRAS transcription factors in sacred lotus (*Nelumbo nucifera*)

**DOI:** 10.7717/peerj.2388

**Published:** 2016-08-31

**Authors:** Yu Wang, Shenglu Shi, Ying Zhou, Yu Zhou, Jie Yang, Xiaoqing Tang

**Affiliations:** 1 College of Horticulture, Nanjing Agricultural University, Nanjing, Jiangsu, China; 2 Institute of Food Crops, Jiangsu Academy Agricultural Sciences, Nanjing, Jiangsu, China

**Keywords:** Genome-wide, GRAS, Nelumbo nucifera, Expression profile, Phylogeny, Sacred lotus

## Abstract

The GRAS gene family is one of the most important plant-specific gene families, which encodes transcriptional regulators and plays an essential role in plant development and physiological processes. The GRAS gene family has been well characterized in many higher plants such as *Arabidopsis*, rice, Chinese cabbage, tomato and tobacco. In this study, we identified 38 GRAS genes in sacred lotus (*Nelumbo nucifera*), analyzed their physical and chemical characteristics and performed phylogenetic analysis using the GRAS genes from eight representative plant species to show the evolution of GRAS genes in *Planta*. In addition, the gene structures and motifs of the sacred lotus GRAS proteins were characterized in detail. Comparative analysis identified 42 orthologous and 9 co-orthologous gene pairs between sacred lotus and *Arabidopsis*, and 35 orthologous and 22 co-orthologous gene pairs between sacred lotus and rice. Based on publically available RNA-seq data generated from leaf, petiole, rhizome and root, we found that most of the sacred lotus GRAS genes exhibited a tissue-specific expression pattern. Eight of the ten PAT1-clade GRAS genes, particularly NnuGRAS-05, NnuGRAS-10 and NnuGRAS-25, were preferentially expressed in rhizome and root. In summary, this is the first *in silico* analysis of the GRAS gene family in sacred lotus, which will provide valuable information for further molecular and biological analyses of this important gene family.

## Introduction

The GRAS genes are plant-specific transcriptional regulators, which have been characterized in many higher plant species. The name GRAS is an acronym of the first three functionally characterized members in this family, including the gibberellic acid insensitive (GAI), repressor of *ga1-3* (RGA), and scarecrow (SCR) (Bolle, 2004). The GRAS proteins contain a variable N-terminal region and a highly conserved C-terminal region with five different sequence motifs in the following order: leucine heptad repeat I (LHR I, LRI), VHIID motif, leucine heptad repeat II (LHR II, LRII), PFYRE motif and SAW motif (Pysh et al., 1999). The GRAS proteins are usually composed of 400–770 amino acid residues (Bolle, 2004). Much research has shown that LRI, VHIID, LRII motif or the whole LRI-VHIID-LRII pattern might function as one of the DNA-binding or protein-binding regions during interaction between GRAS and other proteins. Functions of the PFYRE and SAW motifs are still not very clear, while the highly conserved residues in the PFYRE and SAW motifs indicate that these motifs are potentially related to the structural integrity of the GRAS proteins (Sun et al., 2011). In fact, the order of the conserved domains in all GRAS proteins are similar, and the regulatory specificity of the GRAS proteins seem to be determined by the length and amino acid sequences of their variable amino-termini (Tian et al., 2004). Based on studies on the model plants *Arabidopsis* and rice, members of the GRAS gene family are divided into eight distinct branches, namely LISCL, PAT1, SCL3, DELLA, SCR, SHR, LS and HAM, which are named after a common feature or one of their members (Hirsch & Oldroyd, 2009). However, another phylogenetic analysis grouped the *Arabidopsis* GRAS proteins into ten branches, including LISCL, AtPAT1, AtSCL3, DELLA, AtSCR, AtSHR, AtLAS, HAM, AtSCL4/7, DLT (Sun et al., 2011). In another study, the GRAS genes can be divided into at least 13 branches (Liu & Widmer, 2014). The identification of GRAS members in different species was also slightly different among studies. There were 32 to 34 genes identified for sacred lotus in this study. In addition, 57 and 60 were identified as GRAS genes in rice in two reports (Liu & Widmer, 2014; Tian et al., 2004). In addition, there were 21, 46, 48, 53 and 106 GRAS transcription factors identified in tobacco (Chen et al., 2015), *Prunus mume* (Lu et al., 2015), Chinese cabbage (Song et al., 2014), tomato (Huang et al., 2015) and *Populus* (Liu & Widmer, 2014), respectively.

Members of the GRAS gene family have diverse functions and exert important roles in plant development and physiological processes, such as in gibberellin acid (GA) signal transduction, root development, axillary shoot development, phytochrome signal transduction and transcriptional regulation in response to abiotic and biotic stresses. For example, the phosphomimic/de-phosphomimic RGA, a representative DELLA protein of the GRAS protein family in *Arabidopsis* regulates different bioactivities in GA signaling (Wang et al., 2014). *AtSCL3* plays an essential role in integrating multiple signals during root cell elongation in *Arabidopsis* (Heo et al., 2011). The gene *MONOCULM 1* (*MOC1*) which is highly related to rice tillering has been cloned. As a result of a defect in the formation of tiller buds, the *moc1* mutant plants contain no tillers except a main culm (Li et al., 2003). As a member of the PAT1 branch, the *Arabidopsis* Scarecrow-like 13 (AtSCL13) is a positive regulator of continuous red light signals downstream of phytochrome B (phyB), it also regulates phytochrome A (phyA) signal transduction in a phyB-independent way according to genetic evidences (Torres-Galea et al., 2006). With increasing the activity of two stress-responsive enzymes *α*-amylase and Superoxide dismutase (SOD) in transgenic seedlings, transgenic *Arabidopsis* plants overexpressing *PeSCL7* from *Populus euphratica* Oliv exhibited enhanced tolerance to salt and drought stress due to increased activity of stress-responsive enzymes in the transgenic seedlings (Ma et al., 2010).

Sacred lotus (*Nelumbo nucifera* Gaertn) is a perennial aquatic herb with large showy flowers. It is widely distributed in Australia, China, India, Iran and Japan (Mukherjee et al., 2009). Sacred lotus has been cultivated as a crop in Far-East Asia for over 3,000 years. This plant is widely used for food (edible rhizomes, seeds and leaves) and medicine, and also plays an vital role in cultural and religious activities (Shen-Miller, 2002). The individual parts of sacred lotus, including leaf, stamen, stem, rhizome and seed, have various medicinal properties. For example, the embryos of seeds are used as traditional Chinese medicine in the treatment of nervous disorders, high fevers (with restlessness), insomnia and cardiovascular diseases (e.g., hypertension, arrhythmia). The leaves are mainly used for clearing heat, cooling blood, removing heatstroke and stopping bleeding. The flowers are useful in treating premature ejaculation, bloody discharges and abdominal cramps (Mukherjee et al., 2009).

The GRAS family genes have been well characterized in several plant species, but they have not been investigated in sacred lotus. In 2013, the China Antique variety of the sacred lotus had its complete genome sequenced and the draft genome was released. The final assembly covers 86.5% of the estimated 929 Mbp total genome size, and the genome was well-assembled with a scaffold N50 of 3.4 Mbp. In the recent assembly, a total of 26,685 genes were annotated in the sacred lotus genome study, of which 4,223 represented a minimum gene set for vascular plants by comparisons of the available sequenced genomes (Ming et al., 2013). The release of the whole genome sequence of sacred lotus enabled us to conduct genome-wide identification and comparative analysis of the GRAS gene family in this plant. In this study, we identified 38 GRAS genes in sacred lotus and constructed a phylogenetic tree of the GRAS genes from eight plant species. Also, we analyzed the gene structure and conserved motifs of the sacred lotus GRAS genes, performed comparative analysis for the GRAS genes from *Arabidopsis*, rice and sacred lotus. Furthermore, expression profiles of the sacred lotus GRAS genes were investigated in four different tissues using publically available RNA-seq data. This study provided essential information for the GRAS family genes in sacred lotus and enhanced our understanding of the GRAS family genes in plants.

## Materials and Methods

### Identification and characterization of the sacred lotus GRAS genes

The sacred lotus genome sequence, the annotated sacred lotus gene and protein sequences were downloaded from Lotus-DB (http://lotus-db.wbgcas.cn/, v1.0) (Wang et al., 2015). The newest HMM model for the GRAS transcription factor gene family named PF03514.11 was downloaded from the Pfam database (http://pfam.xfam.org/) (Eddy, 2011; Finn et al., 2014). The HMMER software was employed in searching for GRAS proteins in the entire protein dataset of sacred lotus with a cut-off E-value of 1e^−5^ using PF03514.11 as a query. The identified potential GRAS proteins were manually checked given their presence of the motifs essential for a protein to be defined as GRAS and all of them were retained for further analysis. The coding sequences and the genomic sequences of the identified sacred lotus GRAS genes were obtained in accordance with the GFF3 file specification. The genomic position of each GRAS protein on the assembled mega scaffolds of sacred lotus was also obtained based on the GFF3 file.

### Phylogenetic analysis of the GRAS genes

Eight representative species were selected for comparative analysis of their GRAS proteins. The genome sequences of spruce (*Picea abies*), the first available gymnosperm genome assembly (Nystedt et al., 2013), was collected from the Congenie Website (http://congenie.org/). The annotated proteins of algae (*Chlamydomonas reinhardtii*), moss (*Physcomitrella paten*), fern (*Selaginella moellendorffii*), columbine (*Aquilegia coerulea*), *Arabidopsis thaliana*, and rice (*Oryza sativa*) were downloaded from the Pfam database (v10) (Goodstein et al., 2012). The GRAS genes in these species were identified using the aforementioned method. No GRAS gene was identified in algae. Given that there were two GRAS domains in two rice genes (LOC_Os12g04370, LOC_Os11g04570), only the best hit domain for each of them was selected for phylogenetic tree construction to avoid redundant amino acids.

The ClustalX2 program was used to generate the multiple sequence alignments of the GRAS proteins with the Gonnet protein weight matrix (Larkin et al., 2007). The MEGA program (v6.06) was employed to construct a maximum-parsimony phylogenetic tree using the JTT model with 500 bootstrap replicates (Tamura et al., 2013). All sites in each of the GRAS proteins were involved in the phylogenetic tree construction. To clearly distinguish the genes in this phylogenetic tree, the terms of “moss” and “fern” were added as prefixes to indicate they were genes from *Physcomitrella paten* and *Selaginella moellendorffii*, while the common names were used as prefixes for other species. The frequency of each divergent branch was displayed if its value was higher than 50%. The Adobe Illustrator software was used to clearly show the GRAS branches after classification of all proteins based on the known background information.

### Gene structure and motif analysis

The gene structure was analyzed using the Gene Structure Display Server tool (http://gsds.cbi.pku.edu.cn/, v2.0) (Hu et al., 2015). The UTR sequences were gathered and were displayed in the final gene structure. The MEME software (http://meme-suite.org/doc/download.html, v4.11.0) was used to search for motifs in all 38 sacred lotus GRAS proteins with a motif window length from 6 bp to 50 bp (Bailey et al., 2009). The default number of motifs to be found was set to 10, and these motifs were allowed to be distributed for any number of repetitions.

### Identification of orthologous and paralogous genes

OrthoMCL (v2.0.3) (Li, Stoeckert & Roos, 2003) was used to search for orthologous, co-orthologous and paralogous genes in sacred lotus, *Arabidopsis*, and rice using the entire GRAS protein sequences. We ran ortholog analysis using OrthoMCL with an E-value of 1e^−5^ for all-against-all BLASTP alignment, and a match cut-off value of 50. The orthologous and paralogous relationships were gathered, and displayed using the Circos software (http://circos.ca/) (Krzywinski et al., 2009).

### Analysis of the GRAS gene expression in different tissues

Transcriptome data of leaf, petiole, rhizome internode and root of sacred lotus have been previously generated and processed. The FPKM (fragments per kilobase per million measure) representing the gene expression level of each sacred lotus gene was available in Lotus-DB (http://lotus-db.wbgcas.cn/genome_download/transcript/). We retrieved the FPKM results of all sacred lotus GRAS genes from Lotus-DB and displayed the results using the HemI program (http://hemi.biocuckoo.org/) with the maximum distance similarity metric (Deng et al., 2014).

## Results and Discussion

### Genome-wide identification of GRAS genes in sacred lotus

A total of 38 distinct GRAS transcription factors were identified from the sacred lotus genome (Table S2). We named these GRAS candidates NnuGRAS-01 up to NnuGRAS-38 based on their E-value ranking (Table 1 and Table S2). The length of GRAS proteins varied greatly, ranging from 74 amino acids (aa) to 807 aa. It was noteworthy that the minimum length of a typical GRAS domain is about 350 amino acids (Liu & Widmer, 2014). Based on this criterion, several of the 38 GRAS proteins that we identified in sacred lotus were exceptional. For example, the number of amino acids of NNU_15540-RA, NNU_11453-RA and NNU_26501-RA was 97, 74 and 168, respectively. The average length of coding sequences (CDS) of the GRAS genes (1,544 bp) was longer than the average of all sacred lotus genes (1,135 bp). Analysis revealed that the GRAS genes of sacred lotus contained longer exons and smaller introns.

**Table 1:** Classification and characterization of the sacred lotus GRAS genes.

ID	Name	Branch	HMM E-value	No. of nucleotides	No. of aa	Molecular weight	pI	Formula	Instability index		Aliphatic	GRAVY
NnuGRAS-01	NNU_15494-RA	SCL3/28	3.70E–140	1,434	477	53252.1	5.76	C_2366_H_3773_N_647_O_706_S_21_	54.51	Unstable	99.01	−0.132
NnuGRAS-02	NNU_17397-RA	DELLA	7.30E–139	1,833	610	66855.9	5.4	C_2939_H_4636_N_818_O_902_S_31_	53.52	Unstable	83.38	−0.222
NnuGRAS-03	NNU_14168-RA	SCL3/28	1.30E–137	1,788	488	54286.4	6.04	C_2414_H_3856_N_660_O_716_S_22_	54.16	Unstable	99.98	−0.097
NnuGRAS-04	NNU_20476-RA	PAT1	1.20E–136	6,387	586	64,924	5.59	C_2842_H_4425_N_791_O_891_S_31_	53.55	Unstable	78.94	−0.322
NnuGRAS-05	NNU_16008-RA	PAT1	2.10E–136	4,156	583	64651.4	5.27	C_2836_H_4393_N_779_O_897_S_28_	52.78	Unstable	78.66	−0.318
NnuGRAS-06	NNU_07942-RA	PAT1	6.30E–135	1,611	536	59725.6	4.92	C_2608_H_4118_N_710_O_828_S_33_	43.73	Unstable	78.26	−0.327
NnuGRAS-07	NNU_11308-RA	PAT1	1.00E–131	4,409	548	61113.5	4.95	C_2684_H_4244_N_728_O_834_S_33_	37.54	Stable	83.49	−0.272
NnuGRAS-08	NNU_03991-RA	DELLA	2.60E–129	4,329	593	64180.3	5.1	C_2815_H_4428_N_778_O_886_S_26_	53.2	Unstable	81.32	−0.235
NnuGRAS-09	NNU_26305-RA	DELLA	3.60E–128	1,626	541	59193.2	5.22	C_2630_H_4110_N_720_O_790_S_23_	51.23	Unstable	92.53	−0.084
NnuGRAS-10	NNU_16483-RA	PAT1	4.70E–128	7,898	662	74350.6	5.66	C_3250_H_5105_N_927_O_1014_S_30_	44.32	Unstable	77.95	−0.412
NnuGRAS-11	NNU_21567-RA	DELLA	7.50E–127	1,608	535	58522.3	5.32	C_2585_H_4044_N_720_O_784_S_24_	49.26	Unstable	90.13	−0.096
NnuGRAS-12	NNU_08209-RA	SCL3/28	2.30E–126	2,185	683	75837.6	5.63	C_3330_H_5272_N_948_O_1030_S_24_	61.82	Unstable	83.66	−0.352
NnuGRAS-13	NNU_20591-RA	LS	7.30E–126	1,245	414	45,901	6.3	C_2041_H_3207_N_581_O_606_S_10_	47.77	Unstable	94.78	−0.117
NnuGRAS-14	NNU_16619-RA	SCR	1.60E–124	4,303	784	85555.9	6.06	C_3759_H_5920_N_1072_O_1176_S_19_	57.82	Unstable	82.9	−0.338
NnuGRAS-15	NNU_08928-RA	PAT1	2.60E–123	2,720	550	61,805	6.61	C_2723_H_4317_N_773_O_832_S_19_	44.49	Unstable	87.24	−0.345
NnuGRAS-16	NNU_03584-RA	SCL9	7.00E–119	2,835	746	84235.5	5.29	C_3705_H_5803_N_1037_O_1160_S_25_	53.45	Unstable	77.53	−0.537
NnuGRAS-17	NNU_18761-RA	SCL9	2.70E–118	2,223	627	70849.7	6.01	C_3138_H_4930_N_892_O_959_S_11_	82.07	Unstable	82.07	−0.537
NnuGRAS-18	NNU_08634-RA	PAT1	8.00E–118	5,833	532	58264.3	5.54	C_2495_H_3985_N_743_O_809_S_29_	49.85	Unstable	70.6	−0.462
NnuGRAS-19	NNU_01236-RA	SCR	1.90E–113	1,365	454	49903.5	5.85	C_2197_H_3497_N_631_O_671_S_13_	56.62	Unstable	94.36	−0.211
NnuGRAS-20	NNU_05389-RA	SCL4/7	1.40E–108	3,329	611	67346.8	5.23	C_3000_H_4645_N_815_O_915_S_18_	53.84	Unstable	78.27	−0.325
NnuGRAS-21	NNU_19189-RA	SHR	7.80E–106	1,500	499	56052.4	5.28	C_2461_H_3782_N_686_O_777_S_20_	49.41	Unstable	67.68	−0.411
NnuGRAS-22	NNU_14510-RA	HAM	2.10E–105	2,424	807	87078.9	5.75	C_3863_H_6027_N_1077_O_1182_S_19_	56.65	Unstable	80.62	−0.299
NnuGRAS-23	NNU_17205-RA	SCL4/7	2.10E–104	1,821	606	66470.6	4.85	C_2951_H_4568_N_792_O_918_S_20_	49.03	Unstable	79.72	−0.285
NnuGRAS-24	NNU_18273-RA	HAM	5.30E–102	2,352	783	84714.6	5.85	C_3765_H_5869_N_1047_O_1138_S_22_	57.71	Unstable	81.61	−0.265
NnuGRAS-25	NNU_04162-RA	PAT1	7.80E–100	993	330	36,648	6.4	C_1588_H_2554_N_462_O_483_S_25_	39.75	Stable	78.58	−0.269
NnuGRAS-26	NNU_22900-RA	SHR	1.10E–93	1,326	441	49603.4	5.06	C_2179_H_3457_N_589_O_681_S_25_	43.42	Unstable	84.42	−0.223
NnuGRAS-27	NNU_02726-RA	SHR	4.60E–92	1,314	437	49356.8	5.03	C_2160_H_3379_N_599_O_672_S_27_	46.58	Unstable	77.21	−0.343
NnuGRAS-28	NNU_13719-RA	SHR	2.70E–91	1,374	457	50,818	5.58	C_2274_H_3536_N_616_O_665_S_21_	46.46	Unstable	89.65	−0.048
NnuGRAS-29	NNU_16238-RA	SHR	4.90E–89	1,305	434	48077.9	5.21	C_2145_H_3353_N_575_O_638_S_21_	48.31	Unstable	92.37	0.005
NnuGRAS-30	NNU_05428-RA	HAM	2.90E–88	1,650	549	61261.3	5.84	C_2735_H_4208_N_750_O_810_S_22_	57.5	Unstable	80.35	−0.165
NnuGRAS-31	NNU_08924-RA	SHR	6.40E–86	1,347	448	50029.1	6.23	C_2226_H_3468_N_616_O_652_S_23_	43.81	Unstable	86.88	−0.129
NnuGRAS-32	NNU_04825-RA	SCR	4.00E–85	1,518	442	50581.1	6.05	C_2225_H_3520_N_640_O_681_S_14_	40.46	Unstable	86.06	−0.524
NnuGRAS-33	NNU_16490-RA	SHR	1.20E–84	1,362	453	50501.8	5.92	C_2240_H_3512_N_622_O_657_S_26_	43.89	Unstable	91.04	−0.094
NnuGRAS-34	NNU_26501-RA	SCL4/7	2.30E–41	507	168	19260.9	5.49	C_867_H_1357_N_229_O_257_S_5_	48.5	Unstable	97.44	−0.175
NnuGRAS-35	NNU_03990-RA	PAT1	1.50E–30	6,791	338	38985.3	9.03	C_1732_H_2723_N_483_O_518_S_12_	60.74	Unstable	74.47	−0.695
NnuGRAS-36	NNU_15540-RA	SCL4/7	5.70E–22	294	97	10979.6	4.95	C_488_H_793_N_129_O_151_S_3_	34.28	stable	103.51	−0.167
NnuGRAS-37	NNU_11453-RA	HAM	4.00E–13	225	74	8400.7	5.16	C_384_H_601_N_97_O_108_S_3_	64.4	Unstable	108.11	0.264
NnuGRAS-38	NNU_24984-RA	PAT1	7.10E–06	4,774	441	48806.2	9.12	C_2245_H_3507_N_561_O_609_S_22_	42.85	Unstable	105.01	0.482

Physical and chemical characteristics of all 38 sacred lotus GRAS proteins, including number of amino acids, molecular weight, theoretical pI, formula, instability index, aliphatic index and GRAVY, were analyzed and summarized in Table S2. The average value of theoretical pI was 5.0, suggesting that most proteins were acidic. Only two proteins, NnuGRAS-35 and NnuGRAS-38, with values of theoretical pI at 9.03 and 9.12, respectively, were considered to be alkaline. Three GRAS proteins with an instability index less than 40 were classified as stable and the rest were classified as unstable. The average aliphatic index of all proteins was 85.8, ranging from 67.68 to 108.11. We found that most sacred lotus GRAS proteins contained numerous aliphatic amino acids. The GRAVY scores of all proteins, except NnuGRAS-29 (0.005), NnuGRAS-37 (0.264) and NnuGRAS-38 (0.482), were less than zero, suggesting that these proteins were hydrophilic. Based on a comparative analysis, the physical and chemical characteristics of the sacred lotus GRAS proteins were in general similar to those of Chinese cabbage (Song et al., 2014).

### The representative gene families in sacred lotus

To shed light to all genes in sacred lotus, the genome-wide identification of all possible genes in gene families were performed. A collection of 16,230 Pfam HMM models was used following abovementioned method. A total of 19,925 sacred lotus genes were identified in 4,032 gene families, and some genes were identified into several families for the boundary of some similar gene families were not totally clear. The Pkinase, PPR (Pentatricopeptide repeat), LRR or RRM (RNA recognition motif), MYB, LRRNT (Leucine rich repeat N-terminal domain), TPR (Tetratricopeptide repeat) and WD40 gene families were identified to contain most members in sacred lotus. Most of these abundant gene families were found to be repeats, transcription factors and zinc-finger containing genes. These data were valuable resources for further characterization of genome-wide identification of gene families in sacred lotus.

### Phylogenetic relationship of the GRAS proteins in *Planta*

To shed insight into the evolution of GRAS genes in the plant kingdom, we analyzed the phylogenetic relationship of the GRAS genes from seven plant species, including moss (*Physcomitrella patens*), fern (*Selaginella moellendorffii*), a gymnosperm (*Picea abies*), a monocot (rice, *Oryza sativa*) and a representative eudicot species (*Arabidopsis thaliana*) and two basal eudicots (*Aquilegia coerulea* and *Nelumbo nucifera*). The *Nelumbo* genus, which was the solely extant genus in the family Nelumbonaceae, was one of the earliest eudicot clades (Li et al., 2014). The columbine was also included in the analysis because it belongs to another primitive family between gymnosperms and Nelumbonaceae. The number of GRAS genes in sacred lotus was 38, which was more than in *Arabidopsis* (32–34), but less than in *Prunus mume* (46), *Solanum lycopersicum* (53) and Chinese cabbage (48) (Huang et al., 2015; Lu et al., 2015; Song et al., 2014; Tian et al., 2004).

**Figure 1:**
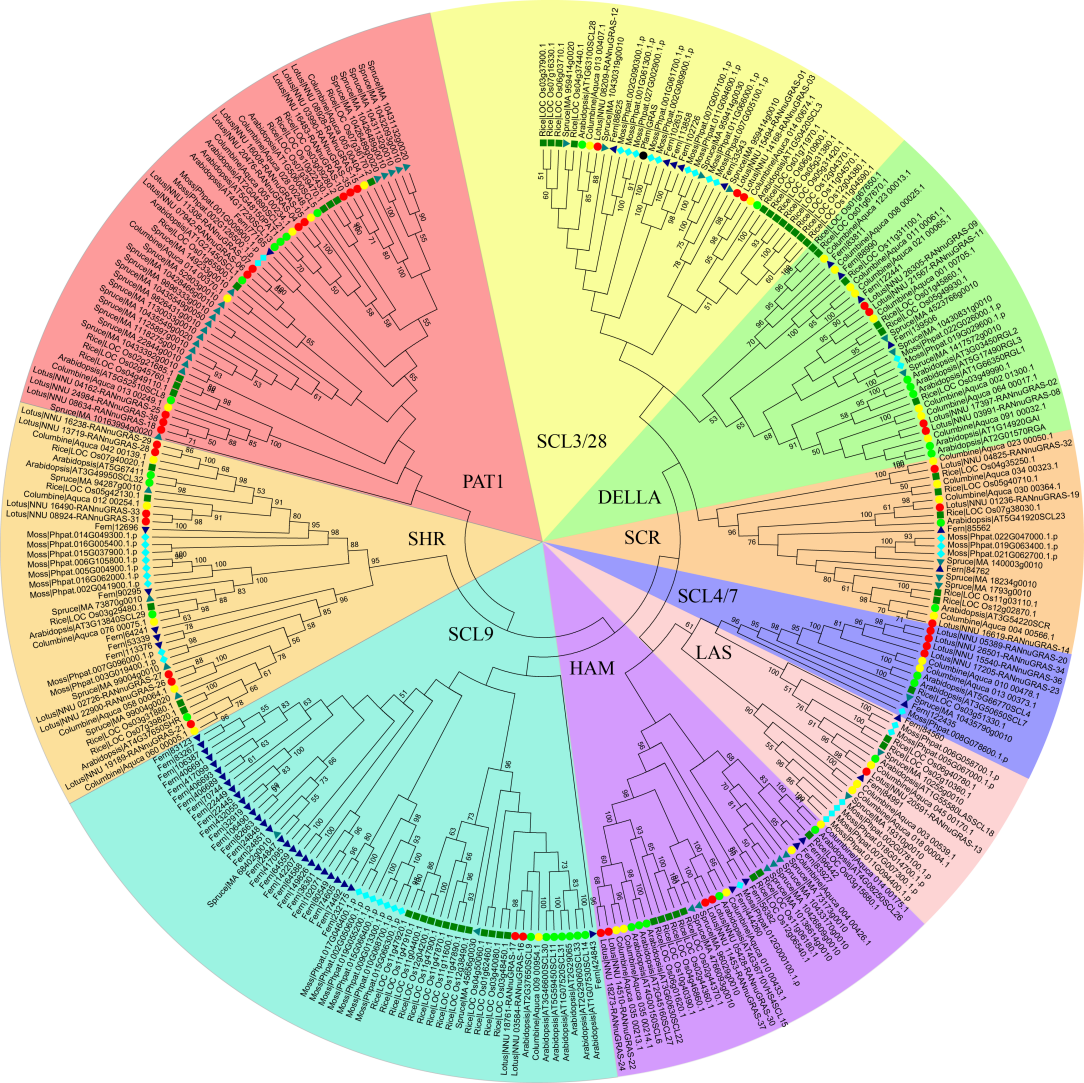
Phylogenetic tree of the GRAS genes from seven plant species, which was constructed based on the GRAS domains using the maximum-parsimony method. Individual species were distinguished by different shapes with different colors.

A previous study showed that the phylogenetic trees based on the full-length sequences or the conserved C-termini of the GRAS proteins were very similar. In both analyses, the GRAS genes were classified into eight branches (Bolle, 2004). The phylogenetic tree of all GRAS genes generated in this study was generally consistent with previous reports. In addition to eight previously reported branches, the new branches SCL3/28 was classified here based on phylogeny. AtSCL8 and AtSCL26 were not classified into any branches for which they were classified into the PAT1 branch and HAM branch due to relative similarity with other members in these two branches. According to the phylogenetic tree, the sacred lotus GRAS genes were distributed in all nine branches, with 4, 3, 4, 1, 4, 2, 7, 10 and 3 in the DELLA, SCR, SCL4/7, LS, HAM, SCL9, SHR, PAT1 and SCL 3/28 branches, respectively (Fig. 1). The SCR and SHR branches contained three (NunGRAS-14, NunGRAS-19 and NunGRAS-32) and seven sacred lotus GRAS proteins, respectively. GRAS genes of the SHR branch have been previously shown to be crucial for the development of root and shoot (Cui et al., 2007). NunGRAS-16 and NunGRAS-17 were classified into the SCL9 branch, whose members have been previously found to regulate the transcription process during microsporogenesis in *Lilium longiflorum* (Morohashi et al., 2003). Ten sacred lotus GRAS proteins were clustered into the PAT1 branch. PAT1 has been shown to be potentially involved in phytochrome. A signal transduction and was found to be localized to the cytoplasm in *Arabidopsis* (Bolle, Koncz & Chua, 2000). The DELLA branch includes four sacred lotus GRAS proteins, including NunGRAS-09, NunGRAS-11, NunGRAS-02 and NunGRAS-08. The *Arabidopsis* GRAS proteins of this branch were found to be associated with negative regulation of GA signaling (Wen & Chang, 2002).

We did not find GRAS genes in the green algae, which in consistent with previous results. The GRAS family transcription factors were found to be first emerged in bacteria and belong to the Rossmann fold methylatransferase superfamily. All bacterial GRAS proteins are likely to function as small-molecule methylases and some of the plant GRAS proteins could have a similar function (Zhang, Iyer & Aravind, 2012). We deduced that the GRAS genes in plants might be originated from bacterial genome. The typical plant GRAS genes first appeared in moss, with 40 members identified in *Physcomitrella patens*. The total number of the GRAS genes in different species fell in a narrow range of 34 to 60; however, the distribution of GRAS genes in different branches was extremely uneven. In some species, the GRAS genes were mainly found in certain branches. For example, 29 of the total 52 fern GRAS genes and 13 of the total 60 rice GRAS genes were classified into the SCL9 branch, while only one columbine (35 in total) and two sacred lotus (38 in total) GRAS genes were found in the SCL9 branch (Fig. 1). The GRAS gene number of *Arabidopsis thaliana* in SCL9 was enlarged into six members, this may result from Gamma, Beta and Alpha whole–genome duplication which will need further research. In addition, nine spruce GRAS genes also accumulated in a sub-branch of the PAT1 branch. As columbine and sacred lotus were closely related to each other based on the phylogenetic analysis, every sacred lotus GRAS gene had close columbine GRAS genes, and most columbine GRAS genes had one or two corresponding GRAS members of sacred lotus except for Aquaca_076_00075.1 and four members in DELLA branch. In one sub-branch of the DELLA branch, there were 2, 4 and 3 fern columbine and rice GRAS genes, respectively, but no moss and spruce GRAS genes. Interestingly, most GRAS genes contained only one typical domain, but two rice genes (LOC_Os12g04370 and LOC_Os11g04570) contained two domains.

**Figure 2:**
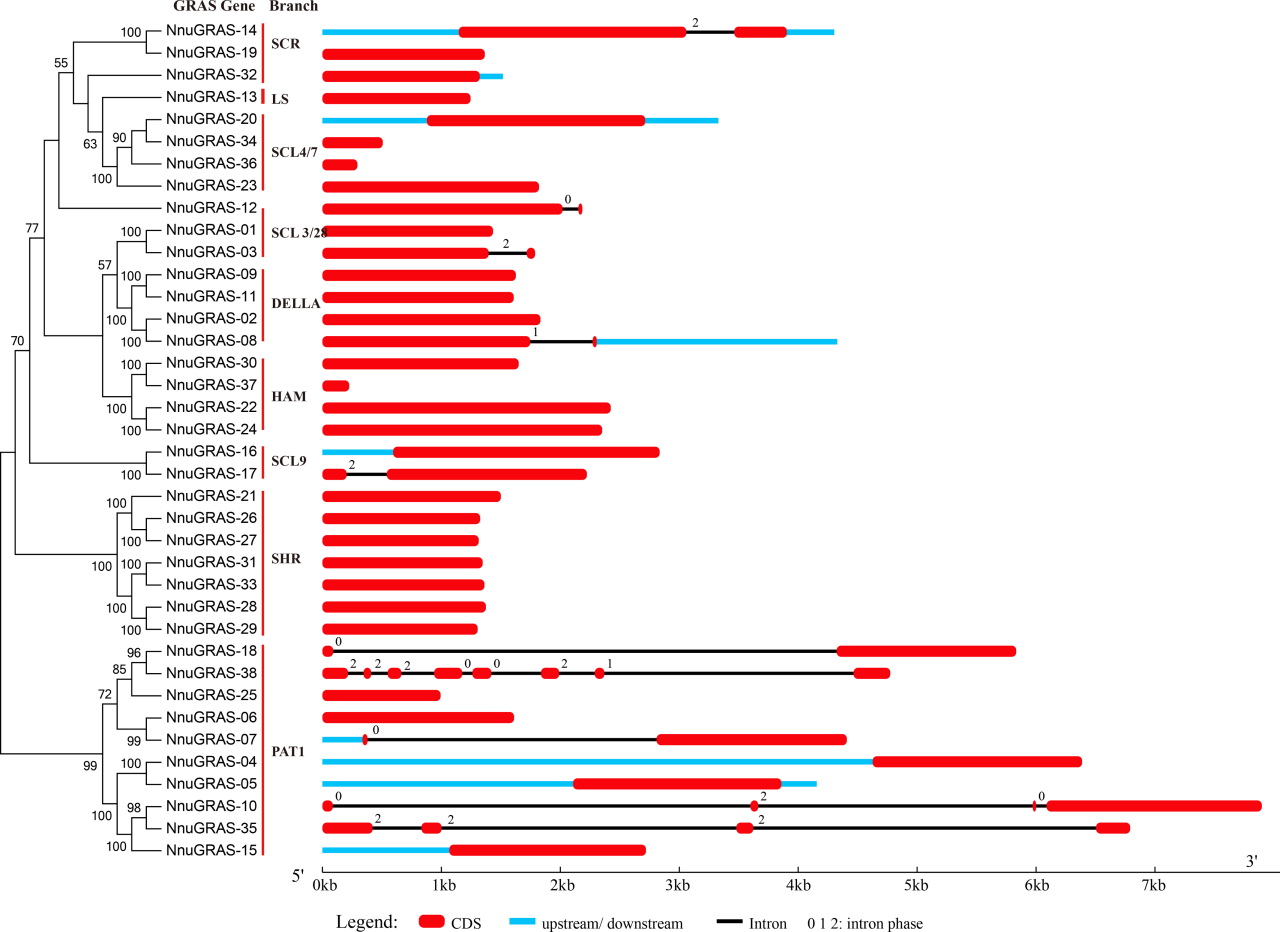
Gene structure of the sacred lotus GRAS genes. Red boxes represent exons, black and blue lines represent introns and UTRs, respectively. The lengths of the exons, introns and UTRs were drawn to scale.

### Gene structure analysis

The structural divergence of exon/intron regions played a crucial role in the evolution of gene families (Bai et al., 2011). To evaluate the possible evolution and diversity of the sacred lotus GRAS genes, we analyzed the number and location of exons, introns and UTRs of each GRAS gene (Fig. 2). The results showed that up to 73.7% (28/38) of the sacred lotus GRAS genes were intronless, which was lower than in *Prunus mume* (82.2%) and tomato (77.4%), but higher than in *Arabidopsis* (67.6%), rice (55%) and *Populus* (54.7%) (Abarca et al., 2014; Liu & Widmer, 2014; Lu et al., 2015; Tian et al., 2004). Only ten of the 38 sacred lotus GRAS genes had introns, ranging from one to seven. Seven genes had a single intron, two genes (NunGRAS-10 and NunGRAS-35) had three introns and NunGRAS-38 had seven introns (Fig. 2). In addition, it was notable that most GRAS members of the same branch generally showed similar exon-intron structures. Most of the paralogous pairs also shared conserved exon-intron structures. However, there were exceptions in the exon-intron structures for different GRAS members of the same branch, such as NunGRAS-14 and -19, NunGRAS-16 and -17 and NunGRAS-18 and -38. These results suggested that one of the genes in these gene pairs might have experienced intron loss or gain events during its evolution process. The intron phase patterns of the GRAS genes in different branches and even in the same branch were totally different, which implied that the relationship of the GRAS genes among different branches was not as close as that observed in other gene families such as the *Squamosa* Promoter Binding Protein (SBP) gene family in *Brassica rapa* and the TIFY gene family in *Arabidopsis* (Bai et al., 2011; Tan et al., 2015). Interestingly, NnuGRAS-38 contained seven introns, while its homolog NnuGRAS-18 contained only one intron, suggesting that even homologs could have experienced gene structure diversification although the possibility of wrong gene annotation could not be ruled out. In addition, NnuGRAS-07, NnuGRAS-10, NnuGRAS-18 and NnuGRAS-35 contained long introns with a length over 4kb, which needs further validation.

### Identification of conserved motifs in the sacred lotus GRAS proteins

**Figure 3:**
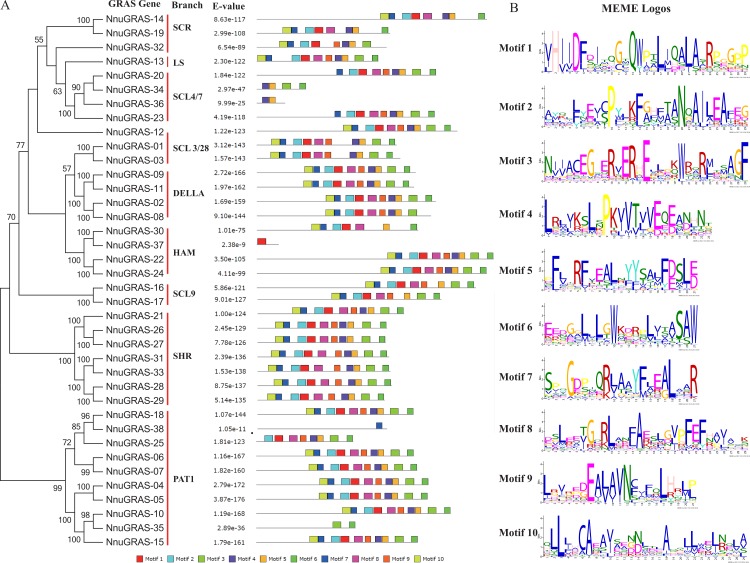
Conserved motifs in the sacred lotus GRAS genes. (A) The phylogenetic tree, the number of conserved motifs and their distribution in each GRAS gene with relative combined *P*-values. (B) Amino acid sequences of each motif. The font size represents the frequency of the respective amino acid.

To analyze the conserved features of the sacred lotus GRAS family proteins, we used the MEME program to identify conserved motifs in all GRAS proteins (Fig. 3A). Surprisingly we found that 23 sacred lotus GRAS proteins contained all 10 motifs, the default number of the MEME analysis, and that only six sacred lotus GRAS proteins contained less than eight motifs. The proteins of the SHR branch was the most representative one of the GRAS gene family with all of them containing 10 motifs. In addition, we found that motifs were more likely to be located in the C-terminal than in the N-terminal, supporting the notion that the C-terminal region of the GRAS proteins was more conserved than the N-terminal region (Pysh et al., 1999). The number of amino acids of these motifs ranged from 21 to 29 (Fig. 3B).

### Identification of orthologous, co-orthologous and paralogous GRAS genes in sacred lotus, *Arabidopsis* and rice

**Figure 4:**
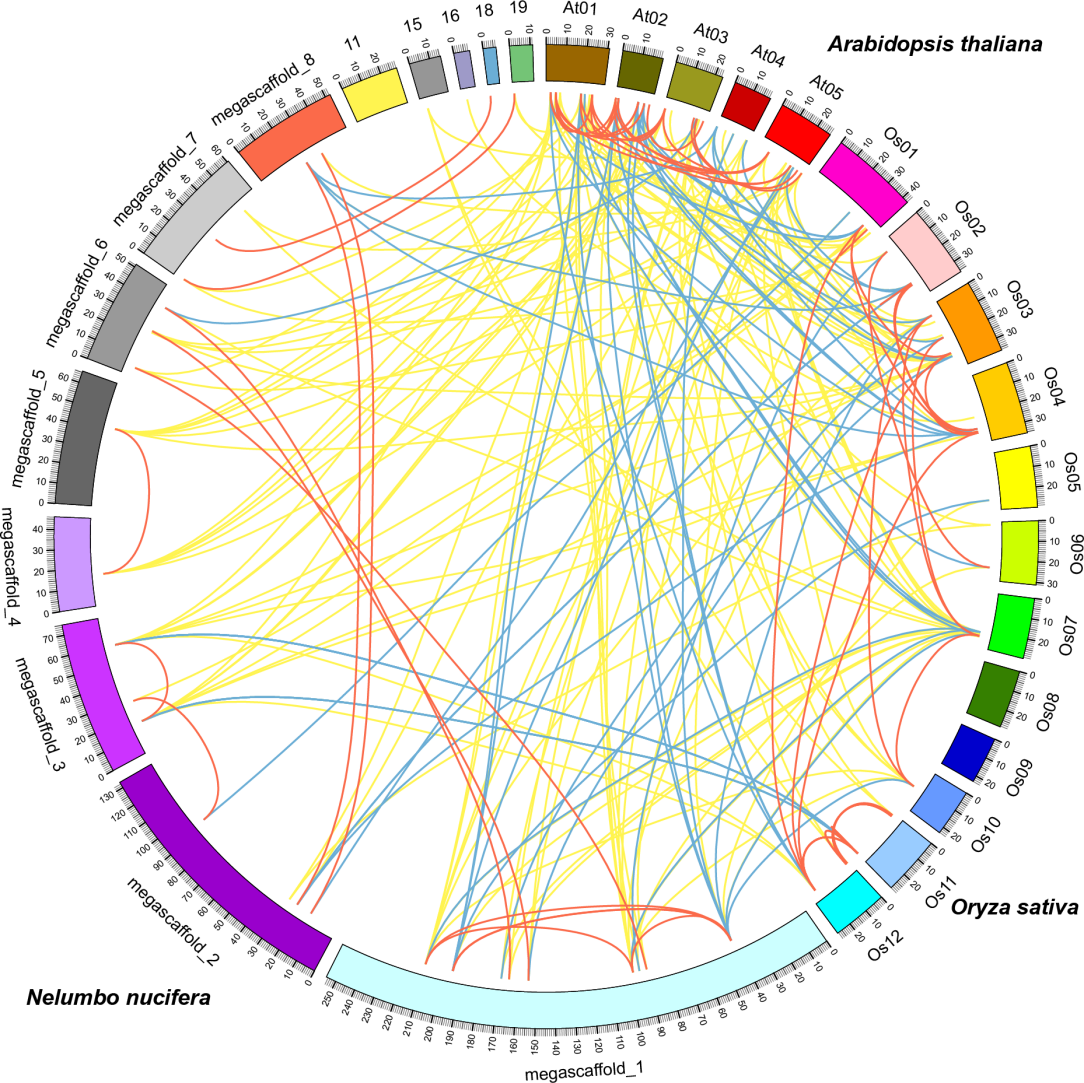
Comparative analysis of the GRAS genes in *Arabidopsis*, rice and sacred lotus. Yellow, blue and red lines indicate orthologous, co-orthologous and paralogous gene pair relationships, respectively.

Comparative analysis identified orthologous, co-orthologous, and paralogous GRAS genes among sacred lotus, *Arabidopsis* and rice using OrthoMCL (Fig. 4). In total, we identified 42 orthologous and nine co-orthologous gene pairs between sacred lotus and *Arabidopsis*, and 35 orthologous and 22 co-orthologous gene pairs between sacred lotus and rice. Between *Arabidopsis* and rice, 29 orthologous and 20 co-orthologous gene pairs were found. In addition, a total of 26 (68.4%) sacred lotus GRAS proteins were found to have corresponding paralogous proteins. This ratio was higher than that in *Arabidopsis* (19, 55.9%) and rice (31, 51.7%) (Table S4).

It was generally considered that orthologous genes have similar gene structure and biological function (Chen et al., 2007). Identification of orthologous genes was crucial for phylogenetic analysis since it could play a role in elucidation of gene and plant evolution. The sacred lotus genome underwent a lineage-specific whole genome duplication (WGD) event about 65 million years ago, which separated it from other eudicots prior to the Gamma genome-triplication event. The lack of the triplication event made the sacred lotus genome an excellent research material bridging eudicots and monocots (Ming et al., 2013). We found that the number of orthologous gene pairs between sacred lotus and *Arabidopsis* were more than that between sacred lotus and rice. In comparison, the number of orthologous GRAS gene pairs between Chinese cabbage and *Arabidopsis* was 52 (Song et al., 2014), a number more than that between sacred lotus and *Arabidopsis*. This result suggested that *Arabidopsis* was genetically close to Chinese cabbage than sacred lotus.

### Expression pattern of the sacred lotus GRAS genes in different tissues

**Figure 5:**
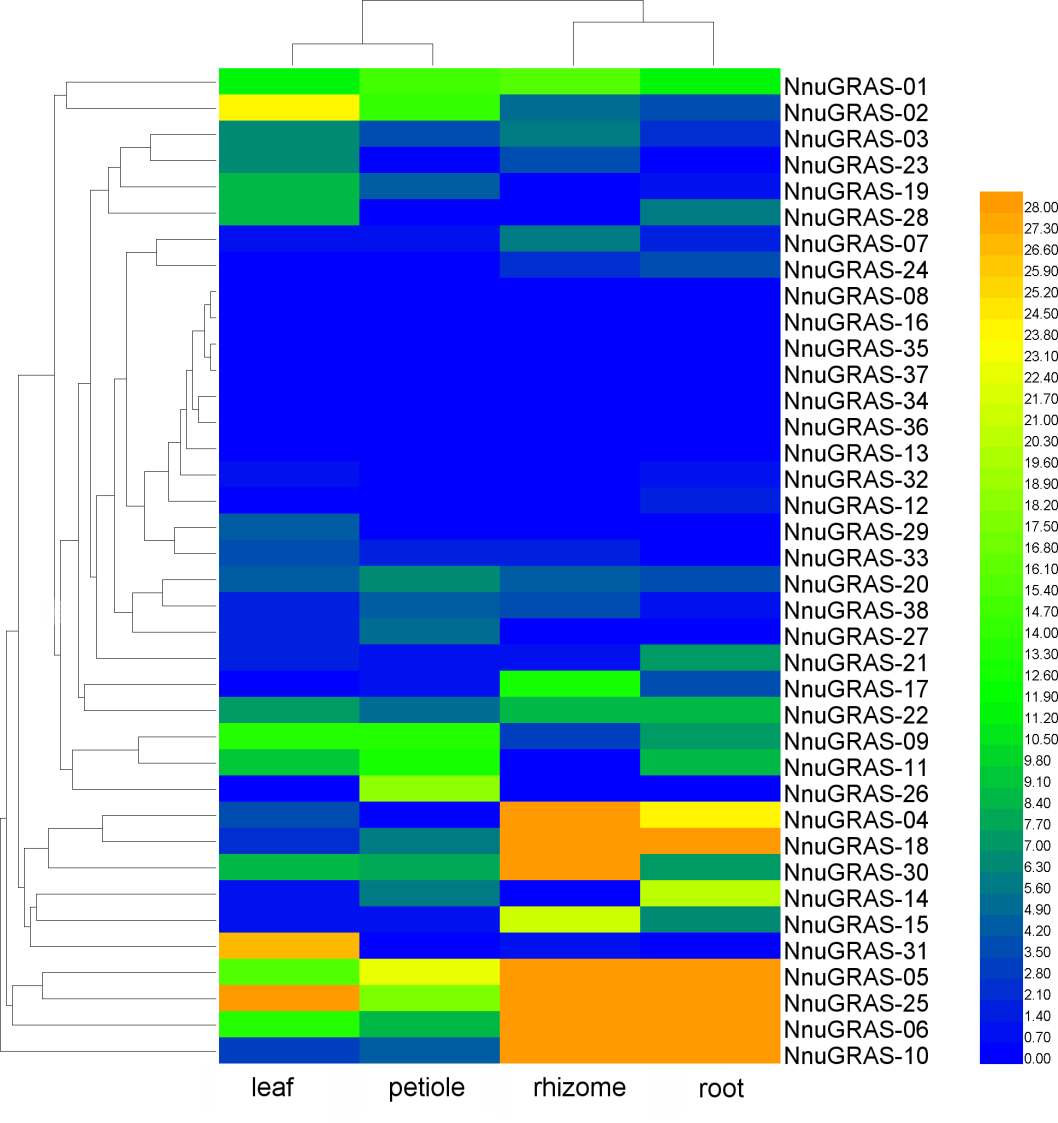
The heat map shows the expression profile of the sacred lotus GRAS genes in four tissues.

We used publically available RNA-seq data of four tissues (leaf, petiole, rhizome and root) to investigate the expression profiles of the sacred lotus GRAS genes (Wang et al., 2015). Based on the maximum distance similarity metric clustering method on two dimensions, we found that the GRAS genes shared a similar expression pattern in leaf and petiole as well as in rhizome and root (Table S2 and Fig. 5). The GRAS genes of some branches exhibited a tissue-specific expression pattern. For example, the GRAS genes of the PAT1 branch seemed to be more abundantly expressed in rhizome and root than in leaf and petiole, especially NnuGRAS-05, NnuGRAS-10, and NnuGRAS-25. This suggests that these genes might be associated with development of the edible sacred lotus rhizome. Further functional characterization of these genes would contribute to increase the production of sacred lotus rhizome through molecular-assisted breeding. Six of the seven GRAS genes of the SHR branch (except NnuGRAS-21) showed a higher expression level in leaf and petiole than in rhizome and root. The members in the SCL 3/28 branch had a similar expression level in the four tissues investigated. The expression levels of NnuGRAS-08, NnuGRAS-16, NnuGRAS-33, NnuGRAS-36 and NnuGRAS-37 were very low in all four tissues (Fig. 5). Some GRAS genes had a unique expression pattern that differentiated them from other members of the same branch. For example, NnuGRAS-26 and NnuGRAS-31 were only relatively highly expressed in petiole and leaf, respectively.

##  Supplemental Information

10.7717/peerj.2388/supp-1 Supplemental Information 1 Supplementary tables

## Additional Information and Declarations

### Data Availability

The following information was supplied regarding data availability: The raw data has been supplied as Supplemental Information.
